# Targeting Toll-Like Receptor 2: Polarization of Porcine Macrophages by a Mycoplasma-Derived Pam2cys Lipopeptide

**DOI:** 10.3390/vaccines9070692

**Published:** 2021-06-23

**Authors:** Giulia Franzoni, Antonio Anfossi, Chiara Grazia De Ciucis, Samanta Mecocci, Tania Carta, Silvia Dei Giudici, Floriana Fruscione, Susanna Zinellu, Guendalina Vito, Simon Paul Graham, Annalisa Oggiano, Bernardo Chessa, Elisabetta Razzuoli

**Affiliations:** 1 Department of Animal Health, Istituto Zooprofilattico Sperimentale della Sardegna, 07100 Sassari, Italy; tania.carta@izs-sardegna.it (T.C.); Silvia.DeiGiudici@izs-sardegna.it (S.D.G.); Susanna.Zinellu@izs-sardegna.it (S.Z.); Annalisa.Oggiano@izs-sardegna.it (A.O.); 2 School of Veterinary Medicine, University of Sassari, 07100 Sassari, Italy; aanfossi@uniss.it; 3 National Reference Center of Veterinary and Comparative Oncology (CEROVEC), Istituto Zooprofilattico Sperimentale del Piemonte, Liguria e Valle d’Aosta, Piazza Borgo Pila 39/24, 16129 Genoa, Italy; chiaragrazia.deciucis@izsto.it (C.G.D.C.); floriana.fruscione@izsto.it (F.F.); Guendalina.vito@izsto.it (G.V.); elisabetta.razzuoli@izsto.it (E.R.); 4 Department of Veterinary Medicine, University of Perugia, 06123 Perugia, Italy; samanta.mecocci@studenti.unipg.it; 5 The Pirbright Institute, Ash Road, Pirbright, Woking GU24 ONF, UK; simon.graham@pirbright.ac.uk

**Keywords:** pig, macrophages, TLR2 agonist, cytokines, surface markers, IFN-α subtypes, toll-like receptors, IL-10

## Abstract

Toll-like receptor 2 (TLR2) ligands are attracting increasing attention as prophylactic and immunotherapeutic agents against pathogens and tumors. We previously observed that a synthetic diacylated lipopeptide based on a surface protein of *Mycoplasma agalactiae* (Mag-Pam2Cys) strongly activated innate immune cells, including porcine monocyte-derived macrophages (moMΦ). In this study, we utilized confocal microscopy, flow cytometry, multiplex cytokine ELISA, and RT-qPCR to conduct a comprehensive analysis of the effects of scalar doses of Mag-Pam2Cys on porcine moMΦ. We observed enhanced expression of activation markers (MHC class I, MHC class II DR, CD25), increased phagocytotic activity, and release of IL-12 and proinflammatory cytokines. Mag-Pam2Cys also upregulated the gene expression of several IFN-α subtypes, p65, NOS2, and molecules with antimicrobial activities (CD14, beta defensin 1). Overall, our data showed that Mag-Pam2Cys polarized porcine macrophages towards a proinflammatory antimicrobial phenotype. However, Mag-Pam2Cys downregulated the expression of IFN-α3, six TLRs (TLR3, -4, -5, -7, -8, -9), and did not interfere with macrophage polarization induced by the immunosuppressive IL-10, suggesting that the inflammatory activity evoked by Mag-Pam2Cys could be regulated to avoid potentially harmful consequences. We hope that our in vitro results will lay the foundation for the further evaluation of this diacylated lipopeptide as an immunopotentiator in vivo.

## 1. Introduction

Toll-like receptors (TLRs) are a group of pattern recognition receptors (PRRs), which play critical roles in initiating host immune defenses. They recognize molecules expressed by pathogens, named pathogen-associated molecular patterns (PAMPs), and endogenous ligands (danger-associated molecular patterns or DAMPs). Some TLRs are located on the cell membrane (TLR1, -2, -4, -5, -6) and recognize microbial lipopolysaccharide or lipopeptides, whereas others are localized intracellularly (TLR3, -7, -8, -9) and bind nucleic acids [1,2]. TLR-ligands are attracting increasing attention as prophylactic and/or therapeutic agents against infectious diseases [3] or in cancer immunotherapy [4]. These molecules target the host rather than pathogens; thus, they are characterized by a broad spectrum of activity and low risk for development of antimicrobial resistance. TLR-agonists can also be used in combination with vaccines as molecular adjuvants, being able to provide a “danger” signal to help induce effective and long-lasting adaptive immune responses [2].

Upon PAMP recognition by TLRs, several intracellular signaling cascades are triggered, which also culminate in inflammasome activation, with consequent inflammatory responses that limit invading pathogens’ progression [5,6]. However, a tight regulation of this process is essential not only to achieve pathogen clearance, but also to avoid pathogenic inflammatory responses [6]. TLR-2 agonists are amongst the TLR-ligands that have shown promise against both pathogens [3] and tumors [7]. The well-characterized macrophage-activating lipopeptide-2 (MALP-2), a lipopeptide originating from *Mycoplasma fermentans* [8], presented remarkable immunomodulatory properties in vivo: its intratracheal administration in rodents led to a recruitment of neutrophils and macrophages to the lungs and increased protection against *Streptococcus pneumonia* [9]. A synthetic analog of MALP-2, S-[2,3-bis(palmitoyl oxy)propyl] cysteine (Pam2Cys) is a potent adjuvant that has been incorporated into many vaccine candidates [10,11,12,13,14,15]. A soluble version of pegylated-Pam2Cys (PEG-Pam2Cys) was intranasally administered in mice in the absence of an antigen [14], leading to a significant recruitment of innate immune cells to the lungs, with subsequent protection to challenge influenza A virus [14]. Most recently, it was reported that intranasal administration of another synthetic TLR2/TLR6 agonist (INNA-051) to ferrets resulted in reduced SARS-CoV-2 shedding in the upper respiratory tract [16].

We previously reported a diacylated lipopeptide, chemically synthesized based on the 14 amino acids following the cysteine immediately downstream the signal peptide of a surface protein of *M. agalactiae* (P48), strongly activated cells of the innate immune system including: ovine neutrophils [17] and monocytes [18], and porcine monocyte-derived macrophages (moMΦ) [19,20]. This diacylated lipopeptide activates cells upon recognition by TLR2, in association with TLR6 [3,17]. We most recently described that this synthetic lipopeptide induced a strong release of IL-12 and proinflammatory cytokines from porcine moMΦ [19,20].

Macrophages are a key component of the innate immune system, responding to both infectious and non-infectious stressors [21]. They are the primary target cell of several viruses threatening the swine industry worldwide, such as the African swine fever virus (ASFV), porcine reproductive and respiratory syndrome virus (PRRSV), and porcine circovirus 2 [22,23,24,25]. New therapeutics, such as immunopotentiators and vaccine adjuvants, might be able to improve the immunological control of these pathogens and reduce the associated economic losses. In addition, pig macrophages resemble human macrophages in many aspects [26,27,28], and overall pig and humans share many immunological similarities [29,30,31], making them an attractive biomedical model [32].

In this study, we deployed an array of techniques to investigate further the effects of Mag-Pam2Cys on porcine macrophages with the aim of informing its potential use as an immunomodulant in vivo.

## 2. Materials and Methods

### 2.1. Animals

Six healthy cross-bred pigs (*Sus scrofa domesticus*), 6–24 months old, were utilized as blood donors. Animals were kept at the Experiment Station of Istituto Zooprofilattico Sperimentale (IZS) of Sardinia (“Surigheddu”, Sassari, Italy). Heparinized blood samples were collected by puncture of the cranial vena cava; all procedures were performed in accordance with the local ethics committee, in agreement with the Guide of Use of Laboratory Animals issued by the Italian Ministry of Health.

### 2.2. Generation of Porcine Monocyte-Derived Macrophages and Activation

MoMΦ cultures were obtained from blood leukocytes, as previously described [20]. In brief, pig leukocytes were first purified from heparinized blood: pig blood was centrifuged at 700× *g* for 30 min at 4 °C without breaks; then, buffy coat was collected, washed three times in red blood cell lysis buffer (distilled water with 0.5 mM Na EDTA, 310 mM NH_4_Cl, 24 mM NaHCO_3_), and finally washed once in PBS. Leukocytes were then cultured in RPMI-1640 supplemented with 10% fetal bovine serum (FBS), 100 U/mL penicillin, and 100 μg/mL streptomycin (complete RPMI, cRPMI), and with 50 ng/mL of recombinant human M-CSF (hM-CSF) (Thermo Fisher Scientific, Waltham, MA, USA), using Petri dishes (2 × 10^7^ leukocytes/mL; 20 mL per Petri dish). After 7 days, non-adherent leukocytes were removed, and adherent moMΦ were detached by gentle scraping using a 25 mL pipette. Cells were centrifuged at 200× *g* for 8 min; supernatants were removed, and moMΦ were finally re-suspended in cRPMI, and seeded in 12-well plates (Greiner CELLSTAR, Sigma-Aldrich, Saint Louis, MO, USA) (1 × 10^6^ live cells per well) or 8-well chamber slides (Thermo Fisher Scientific) (1 × 10^5^ live cells per well). Cells were incubated at 37 °C 5% CO_2_ for a further 24 h before treatment [20].

MoMΦ were left untreated or stimulated with 10 or 100 ng/mL of the TLR2 agonist S-[2–bis(palmitoyl)-propyl]cysteine (Pam2Cys) lipopeptide (Mag-Pam2Cys). Mag-Pam2Cys was chemically synthesized based on the 14 amino acids following the cysteine immediately downstream the signal peptide of a *M. agalactiae* lipoprotein (P48: CGDKYFKETEVDGV) (Espikam, Prato, Italy) [17].

In selected experiments, cells were stimulated with 100 ng/mL of recombinant LPS (lipopolysaccharide from *Escherichia coli* 0111:B4; Sigma-Aldrich) and 100 ng/mL of recombinant porcine IFN-γ (RayBiotech Inc., Norcross, GA, USA) for 24 h, in order to generate moM1, as previously described [20].

In other selected experiments, cells were stimulated with Mag-Pam2Cys (10 or 100 ng/mL) and recombinant porcine IL-10 (20 ng/mL). Recombinant porcine IL-10 (R&D System, Minneapolis, MN, USA) was added simultaneously for 24 h before Mag-Pam2Cys stimulation. Untreated cells were included as controls.

### 2.3. Cell Viability

The impact of Mag-Pam2Cys on moMΦ viability was evaluated using a non-radioactive cytotoxicity assay [33]. In brief, cells were seeded in 12 well plates (1 × 10^6^ live cells per well) and left untreated (negative control) or cultured in the presence of scalar doses of Mag-Pam2Cys (10 and 100 ng/mL). Then, 24 h later, lactate dehydrogenase (LDH) levels in culture supernatants were quantified using the Cytotox 96 Non-Radioactive Cytotoxicity Assay (Promega, Madison, WI, USA), and a lysis solution provided by the manufacturer was used as a positive control, according to the manufacturer’s instruction. Absorbance was read at 492 nm using an Epoch microplate reader (BioTek, Winoosky, VT, USA).

### 2.4. Flow Cytometry

MoMΦ were seeded in 12 well plates (1 × 10^6^ live cells per well) and stimulated with Mag-Pam2Cys, with or without IL-10. Untreated cells were used as a control. Then, 24 h post stimulation, flow cytometry was carried out as previously described [19]. In brief, cells were harvested with PBS with 10 mM EDTA and transferred into 5 mL round bottom tubes (Corning). MoMΦ were washed, resuspended in 0.1 mL per tube, and stained with Zombie Aqua dye (BioLegend, San Diego, CA, USA), and after 30 min of incubation at room temperature, cells were washed with PBS supplemented with 0.5% bovine serum albumin (BSA). Cells were then resuspended in 0.1 mL of PBS, and staining with monoclonal antibodies was performed using mouse anti human CD14-PerCP (Tuk4; Miltenyi Biotec, Bergisch Gladbach, Germany), mouse anti pig MHC class II DR-FITC (2E9/13, Bio-Rad Antibodies, Kidlington, UK), MHC class I (JM1E3, Bio-Rad Antibodies), CD163-PE (2A10/11, Bio-Rad Antibodies), CD169-FITC (MCA2316F, Bio-Rad Antibodies), and CD25 (K231.3B2, Bio-Rad Antibodies). Incubation was performed at 4 °C for 15 min. MHC I and CD25 expression were visualized by subsequent staining with BV421 rat anti mouse IgG1 (A85-1, BD Horizon, BD Biosciences, Franklin Lakes, NJ, USA) for a further 15 min. Both primary and secondary antibody were added at a final volume of 10 μL per tube; their details are reported in Table S1. After washing with PBS supplemented with 2% FBS, cells were resuspended in PBS supplemented with 2 mM EDTA and were analyzed with a FACS Celesta (BD Biosciences). A total of 5000 live moMΦ were acquired. Analysis of data was performed using BD FACS Diva Software (BD Biosciences), by exclusion of doublets, gating on viable moMΦ, and then assessing the staining for surface markers [19].

### 2.5. Impact of Pam2Cys on moMΦ Morphology

MoMΦ were cultured in 8-well chamber slides (for confocal microscopy) or 12-well plates (for flow cytometry), at 1 × 10^5^ or 1 × 10^6^ live cells per well, respectively. Then, 24 h post-activation, Mag-Pam2Cys’ impact on moMΦ phenotype was assessed. For confocal microscopy, moMΦ were fixed with 4% paraformaldehyde and then labelled with Hoechst 33342 and Alexa Fluor 488 conjugated phalloidin (both Molecular Probes, Thermo Fisher Scientific, Rockford, IL, USA) to visualize nuclei or actin cytoskeleton, respectively [34]. Confocal microscopy was performed using a Leica SP5 Confocal Microscope (Leica Microsystem, Wetzlar, Germany) equipped with a 40X Plan-Apo 1.25 NA oil immersion objective. Images were acquired on a format of 1024 × 1024 pixel and were processed with LAS AF Lite software (Leica Microsystem), as previously described [34]. Phase-contrast microscopy images were also acquired: MoMΦ were fixed with 4% paraformaldehyde, washed with PBS, and then images were acquired using an inverted stereo microscope Olympus IX 70 (Segrate, Italy). Furthermore, moMΦ were morphologically evaluated though May–Grunwald–Giemsa staining. In brief, moMΦ were fixed, washed with PBS, and stained using the May–Grünwald–Giemsa method, and images were acquired and recorded using a light microscope (Nikon Eclipse 80i, Tokyo, Japan) equipped with a Nikon Ds-fi1 camera (Tokyo, Japan). Flow cytometry was carried as above described, and forward scatter (FSC) and side scatter (SSC) data were used to determine effects on the dimension and granularity of moMΦ.

### 2.6. Phagocytosis Assay

MoMΦ were cultured in 8-well chamber slides (1 × 10^5^ live cells/well). Then, 24 h after seeding, cells were left untreated or stimulated with Mag-Pam2Cys (10 or 100 ng/mL), and phagocytosis assays were performed 24 h later using pHrodo Red Zymosan A Bioparticles Conjugated (Molecular Probes), following the manufacturer’s instruction. In brief, culture supernatants were removed, replaced with 0.1 mL of pHrodo bioparticles suspension (0.5 mg bioparticles/mL), and incubated for 2 h at either 4 °C (control) or 37 °C for 2 h. MoMΦ were then fixed with 4% paraformaldehyde and labelled with Hoechst 33342 (Molecular Probes) to visualize nuclei. Microscopy was performed as above described, using a Leica SP5 Confocal Microscope, and processing images with LAS AF Lite software [34].

### 2.7. Cytokine Levels Determination

MoMΦ were cultured in 12-well plates (1 × 10^6^ live cells/well) and stimulated with Mag-Pam2Cys (10 or 100 ng/mL) or left untreated. Then, 24 h later, culture supernatants were removed, cleared by centrifugation (2000× *g* for 3 min), and stored at −80 °C until analysis. Levels of IL-1α, IL-1β, L-6, IL-10, IL-12, TNF-α were determined using a Porcine Cytokine/Chemokine Magnetic Bead Panel Multiplex assay (Merck Millipore, Darmstadt, Germany) and a Bioplex MAGPIX Multiplex Reader (Bio-Rad, Hercules, CA, USA), according to the manufacturers’ instructions, as previously described [34].

### 2.8. Gene Expression

Changes in the mRNA expression profile were evaluated as previously described [19,35,36]. In brief, moMΦ were seeded in 12 well plates (1 × 10^6^ live cells/well) and left untreated or stimulated with Mag-Pam2Cys (10 or 100 ng/mL); at selected time points (0, 4, 8, 24 h), culture supernatants were removed, and cells were lysed with buffer RTL (Qiagen, Hilden, Germany). Total RNA was then extracted using the RNeasy Mini Kit (Qiagen) and eluted in 100 µL of ultrapure RNase-free water. Furthermore, 250 ng of purified RNA was used as the template for cDNA synthesis, as previously described [36]. Gene expression was evaluated by RT-qPCR, using primer sets reported in Table S2 [37,38,39,40,41]. Real-time PCR amplification was performed in a CFX96 Real-Time System after the reverse transcription step, with glyceraldehyde 3-phosphate dehydrogenase (GAPDH) as a reference gene [35]. In each sample, the relative expression of the test genes was calculated using the widely adopted 2^−ΔΔCq^ method, with the Cq acronym of the quantification cycle [35].

### 2.9. Statistical Analysis

Experiments were carried out in technical duplicate and repeated with at least three different blood donor pigs. Data were first checked for normality using Minitab (Minitab Inc., Coventry, UK), and then they were graphically and statistically analyzed with GraphPad Prism 8.01 (GraphPad Software Inc., La Jolla, CA, USA). Data normality was determined using the Anderson Darling test; then, data were analyzed using the parametric one-way ANOVA followed by Dunnett’s multiple comparison test or the non-parametric Kruskal–Wallis test followed by a Dunn’s multiple comparison test. In both cases, a *p* value < 0.05 was considered statistically significant; *p* < 0.05 (*), *p* < 0.01 (**), *p* < 0.001 (***).

## 3. Results

The ability of Mag-Pam2Cys, a chemically synthesized TLR2 agonist, to modulate the porcine moMΦ phenotype and functionality was assessed though an integrative analytical approach, spanning flow cytometry, confocal microscopy, multiplex ELISA, and RT-qPCR. The ability of this lipopeptide to affect the macrophage’s responses to the immunosuppressive IL-10 was also evaluated (Figure S1).

### 3.1. Impact of Mag-Pam2Cys on moMΦ Phenotype and Viability

**Figure 1:**
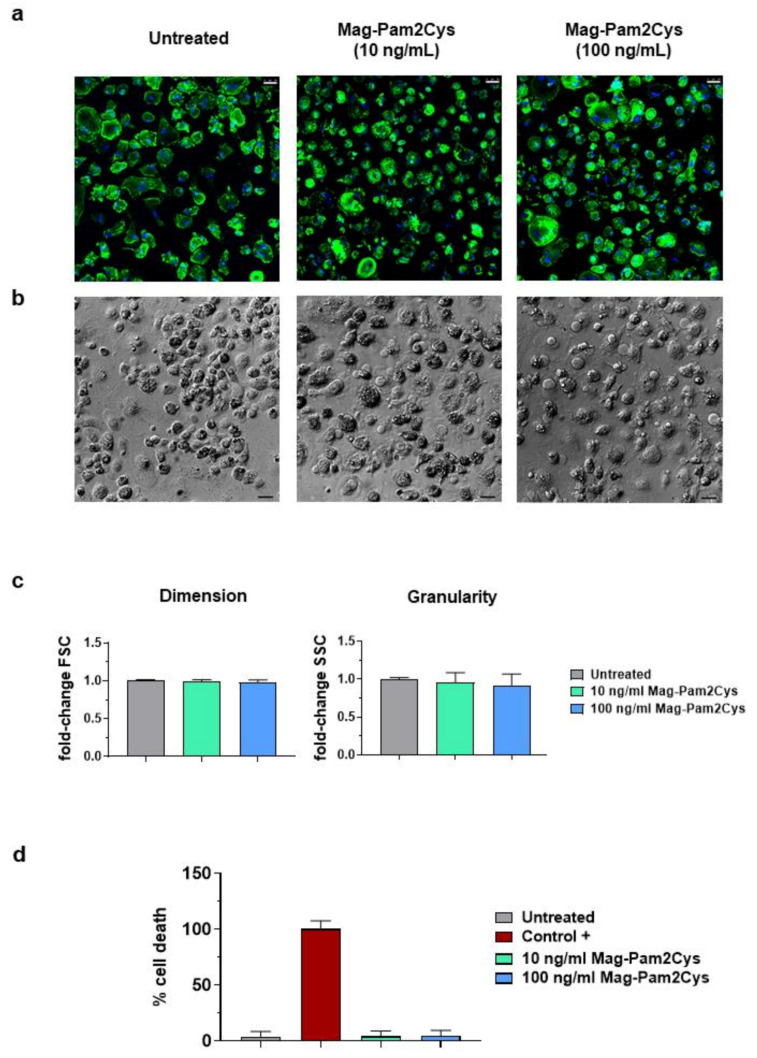
Impact of Mag-Pam2Cys on porcine moMΦ morphology and viability. Porcine moMΦ were stimulated with scalar doses of Mag-Pam2Cys (10 or 100 ng/mL) or left untreated. Then, 24 h post-stimulation, morphology (**a**–**c**) and viability (**d**) were assessed. (**a**) Confocal microscopy observation after nuclei staining with Hoechst 33342 (blue) and cytoskeleton with Alexa Fluor 488-conjugated phalloidin (green), with magnification 40×. Scale bar, 25 µm. (**b**) Phase-contrast microscopy images were acquired using an inverted microscope, with magnification 20×. Scale bar, 25 µm. For both a and b, images of representative moMΦ, one from each condition (untreated, 10 or 100 ng/mL of Mag-Pam2Cys) are presented. (**c**) Changes in dimension and granularity of moMΦ were evaluated by flow cytometry. Forward scatter (FSC) and side scatter (SSC) data are displayed as fold-change relative to untreated moMΦ. (**d**) Mag-Pam2Cys impact on moMΦ viability was assessed using a non-radioactive cytotoxic assay. A lysis solution provided by the manufacturer was used as positive control (Control +). For both Panel (**c**,**d**), mean data for triplicate biological replicates and standard deviation (SD) are shown. Values of stimulated samples were compared to the untreated control using a one-way ANOVA followed by a Dunnett’s multiple comparison test.

MoMΦ were left untreated or stimulated with 10 or 100 ng/mL of Mag-Pam2Cys. Then, 24 h later, their morphology was assessed by confocal microscopy and flow cytometry (Figure 1). Mag-Pam2Cys did not significantly impact moMΦ morphology: Cells presented with a spherical shape with short hairy protrusions on their surface, as observed in our previous study [19], irrespective of the treatment (Figure 1a). No morphological alterations were observed using phase contrast microscopy or May–Grunwald–Giemsa staining (Figure 1b, Figure S2). No changes in cellular dimension or granularity were detected between Mag-Pam2Cys stimulated or untreated moMΦ (Figure 1c). At the same time point post-stimulation (24 h), the effects of Mag-Pam2Cys on moMΦ viability were quantified using a cytotoxicity assay. A lysis solution provided by the manufacturer and untreated moMΦ were used as positive and negative controls, respectively. Mag-Pam2Cys had no appreciable impact on porcine macrophage viability (Figure 1c).

**Figure 2:**
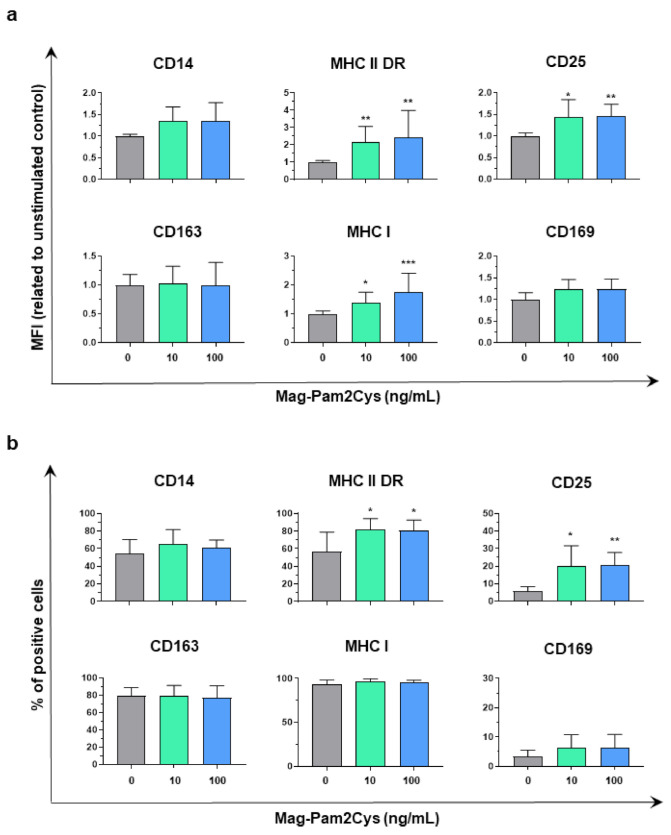
Effect of Mag-Pam2Cys on moMΦ surface marker expressions. Then, 24 h post-stimulation with scalar doses of Mag-Pam2Cys (10 or 100 ng/mL), surface expression of CD14, MHC II DR, CD25, CD163, MHC I, and CD169 were assessed by flow cytometry. In Panel (**a**), mean fluorescence intensity (MFI) data are presented as fold-change relative to the untreated control (moMΦ). In Panel (**b**), percentages of positive cells are shown. For both Panels, mean data and SD from four independent experiment using different blood donor pigs are displayed. Values of Mag-Pam2Cys-treated samples were compared to the untreated control (moMΦ) using a one-way ANOVA followed by a Dunnett’s multiple comparison test. *** *p* < 0.001, ** *p* < 0.01, * *p* < 0.05.

The modulation of several key surface markers by Mag-Pam2Cys was assessed by flow cytometry (Figure 2, Figure S3). Both 10 and 100 ng/mL doses of Mag-Pam2Cys induced an upregulation of the surface expression of MHC I, MHC II DR, and CD25. Statistically significantly upregulation of MHC II DR and CD25 was appreciated in terms of both mean fluorescence intensity (MFI) and percentage of positive cells, and a significant increase in the MFI (but not percentage of positive cells) of the ubiquitously expressed MHC I was observed (Figure 2, Figure S3). No differences were observed between untreated and Mag-Pam2Cys stimulated moMΦ in terms of percentages of CD163^+^ or CD169^+^ cells. Stimulation with this TLR2 agonist induced a slight increase in CD14 expression (MFI), although without statistical significance (Figure 2, Figure S3).

### 3.2. Evaluation of the Effects of Mag-Pam2Cys on moMΦ, Phagocytic Capacity, and Cytokine Responses

**Figure 3:**
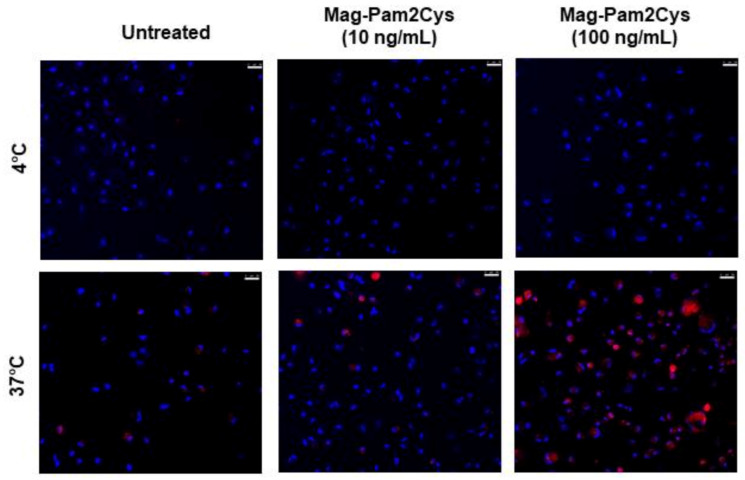
Mag-Pam2Cys’ impact on moMΦ phagocytotic ability. Porcine moMΦ were left untreated or stimulated with scalar doses of Mag-Pam2Cys (10 or 100 ng/mL). At 24 h post-stimulation, a phagocytosis assay was performed using pHrodoTM Red Zymosan A Bioparticles Conjugate (red). Cells were incubated with bioparticles for 2 h, at either 4 °C (control) or 37 °C. Then, nuclei were stained with Hoechst 33342 (blue), and confocal microscopy was performed using a 40× magnification. Images of representative moMΦ, one from each condition (untreated, 10 or 100 ng/mL of Mag-Pam2Cys) are presented. Scale bar, 25 µm.

The effect of Mag-Pam2Cys on moMΦ phagocytotic activity was then monitored using confocal microscopy. At 24 h post-stimulation, an increased number of moMΦ associated with red-labelled bioparticles was observed in the Mag-Pam2Cys-treated condition compared to the untreated control, especially in cells exposed to the higher dose (100 ng/mL) (Figure 3).

**Figure 4:**
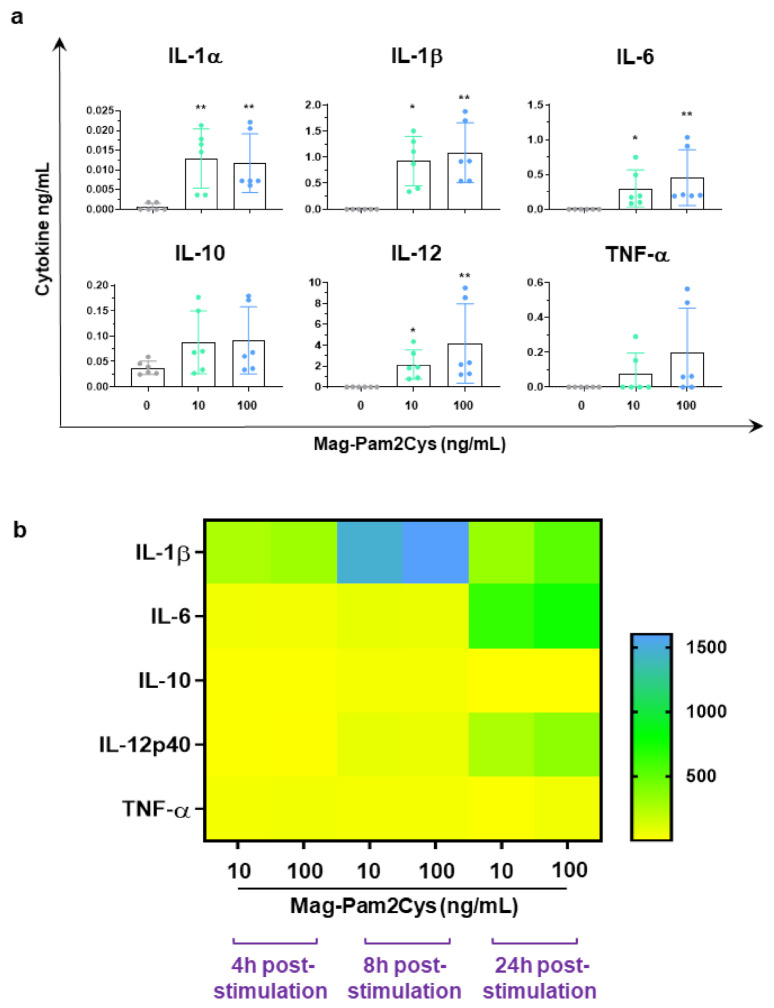
Mag-Pam2Cys’ impact on cytokine induction and release. Porcine moMΦ were left untreated or stimulated with scalar doses of Mag-Pam2Cys (10 or 100 ng/mL). (**a**) At 24 h post-stimulation, levels of IL-1α, IL-1β, IL-6, IL-10, IL-12, TNF-α in culture supernatants were quantified using a multiplex ELISA. Mean data and SD from three independent experiments using different blood donor pigs are presented. Values for Mag-Pam2Cys-stimulated samples were compared to the corresponding untreated control (moMΦ) using a Kruskal–Wallis multiple comparison test; ** *p* < 0.01, * *p* < 0.05. (**b**) At 4, 8, and 24 h post-stimulation, gene expression levels of IL-1β, IL-6, IL-10, IL-12p40, TNF-α genes were determined using RT-qPCR. At each time point, data were normalized on the values of untreated control and expressed as 2^−ΔΔCq^, with ΔCq = Cq (target gene)—Cq (reference gene), and ΔΔCq = ΔCq (Mag-Pam2Cys-stimulated samples)—ΔCq (untreated sample, moMΦ). Heatmap displays mean data from six independent experiments using different blood donor pigs. The colors in the cells represent the relative magnitude of gene expression. The green color represents the average magnitude of gene expression. The yellow color represents the smallest value, and the brightest blue represents the highest value.

At the same time post-stimulation, the cytokine content in culture supernatants was assessed by ELISA. Levels of several proinflammatory cytokines (IL-1α, IL-1β, IL-6, TNF-α) and IL-12 were increased in Mag-Pam2Cys-stimulated cultures (Figure 4a). Low levels of IL-10 were also detected in the culture supernatant of cells exposed to either 10 or 100 ng/mL of Mag-Pam2Cys, although without statistical significance compared to the untreated control (Figure 4a). To investigate moMΦ cytokine response to Mag-Pam2Cys further, gene expression analyses of IL-1β, IL-6, TNF-α, IL-12p40, and IL-10 were performed. MoMΦ were exposed to Mag-Pam2Cys (10 or 100 ng/mL) or left untreated, and at 4, 8, and 24 h post-stimulation, expression analysis of the five selected cytokine genes was performed (Figure 4b). RT-qPCR data revealed that Mag-Pam2Cys induced enhanced expression of IL-1β, IL-6, TNF-α, and IL-12p40 at all the tested time points, in full accordance with the cytokine protein levels detected in moMΦ culture supernatants. Statistically significant increased expression of IL-10 was observed only at 4 and 8 h post-stimulation (Figure 4b, Figure S4).

### 3.3. Comparison of Mag-Pam2Cys and M1-Polarization

**Figure 5:**
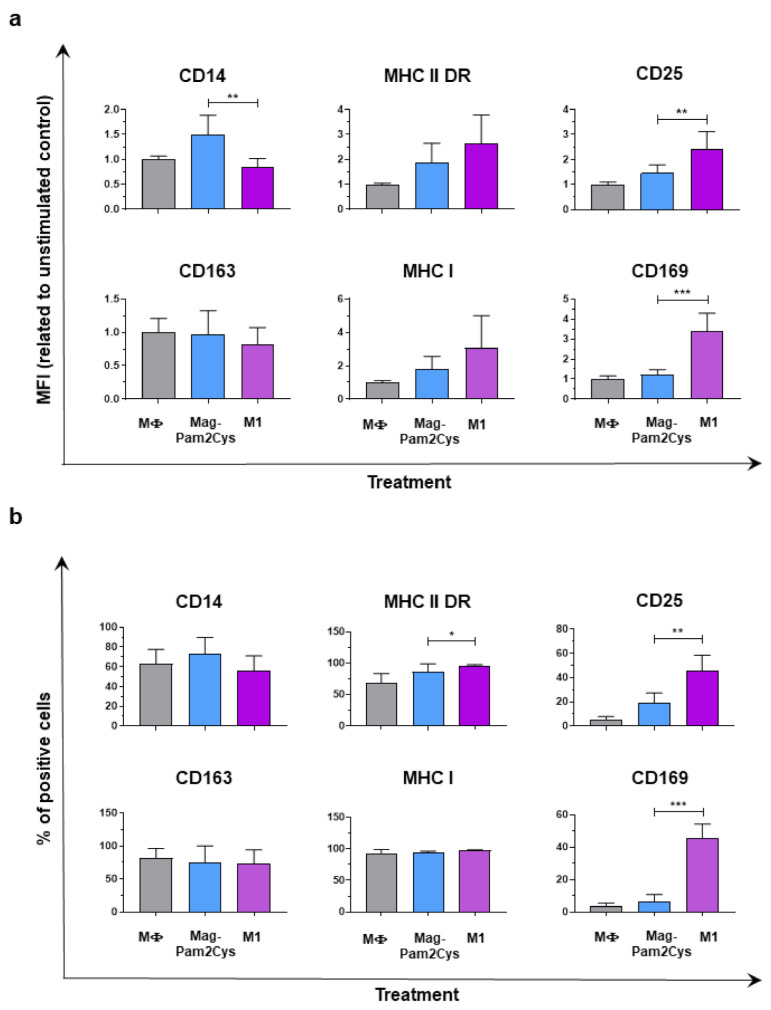
Modulation of moMΦ surface marker expressions by Mag-Pam2Cys and classical M1 polarizing factors. Porcine moMΦ were left untreated (MΦ) or stimulated with Mag-Pam2Cys (100 ng/mL) or IFN-γ + LPS (M1). At 24 h post-stimulation, surface expression of CD14, MHC II DR, CD25, CD163, MHC I, and CD169 were assessed by flow cytometry. In Panel (**a**), mean fluorescence intensity (MFI) data are presented as fold-change relative to the untreated control (moMΦ). In Panel (**b**), percentages of positive cells are shown. For both Panels, mean data and SD from three independent experiments using different blood donor pigs are displayed. Values of Mag-Pam2Cys-treated samples were compared to bona fide moM1 (M1), using a Mann–Whitney test. *** *p* < 0.001, ** *p* < 0.01, * *p* < 0.05.

So far, our data revealed that Mag-Pam2Cys induced an upregulation of the surface expression of MHC I, MHC II DR, and CD25, increased phagocytic activity, and a release of IL-1β, IL-6, IL-12p40, TNF-*α*. These data suggest that Mag-Pam2Cys polarized porcine macrophages toward a M1-like phenotype [34,42]; thus, we compared the impact of the higher dose of Mag-Pam2Cys against the M1 polarizing factors IFN-γ and LPS on porcine macrophages. Differences in the modulation of surface marker expression (MHC I, MHC II, CD25, CD14, CD163, CD169) and cytokine release (IL-1α, IL-1β, IL-6, IL-10, IL-12p40, TNF-*α*) were assessed. MoMΦ stimulated with Mag-Pam2Cys presented a lower expression of CD25 and CD169 compared to moM1, in terms of both mean fluorescence intensity (MFI) and percentage of positive cells (Figure 5). MoM1 were also characterized by a higher expression of both MHC I and MHC II DR, although differences were statistically significant only for percentages of positive MHC II DR cells (Figure 5). Differences were observed in terms of CD14 expression: MoM1 presented a lower expression of this marker compared to macrophages stimulated with Mag-Pam2Cys (Figure 5).

**Figure 6:**
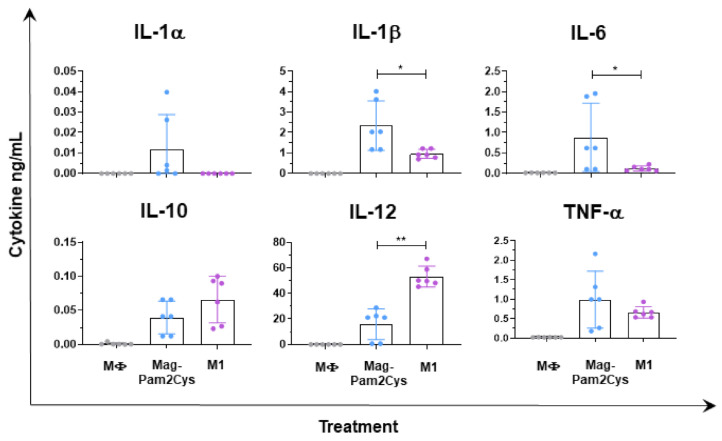
Cytokine release from moMΦ stimulated with Mag-Pam2Cys or IFN-γ and LPS. Porcine moMΦ were left untreated (MΦ) or stimulated with Mag-Pam2Cys (100 ng/mL) or IFN-γ + LPS (M1). At 24 h post-stimulation, levels of IL-1α, IL-1β, IL-6, IL-10, IL-12, TNF-α in culture supernatants were quantified using a multiplex ELISA. Mean data and SD from three independent experiments using different blood donor pigs are displayed. Values of Mag-Pam2Cys-treated samples were compared to bona fide moM1 (M1), using a Mann–Whitney test. ** *p* < 0.01, * *p* < 0.05.

Stimulation of moMΦ with Mag-Pam2Cys or with IFN-γ/LPS resulted in different patterns of cytokine release. MoMΦ stimulated with Mag-Pam2Cys presented a higher release of IL-6, IL-1β, IL-1α compared to moM1, although the latter cytokine was not statistically significant (Figure 6). MoMΦ stimulation with IFN-γ/LPS resulted instead in a higher release of IL-12 compared to 100 ng/mL of MagPam2Cys (Figure 6).

### 3.4. Effect of Mag-Pam2Cys on Key Macrophage Regulatory Gene Expression

To gain a broader view of the effects of Mag-Pam2Cys on moMΦ, additional gene expression analyses were performed. MoMΦ were exposed to Mag-Pam2Cys (10 or 100 ng/mL) or left untreated, and at 4, 8, and 24 h post-stimulation, expression analysis of a further 28 selected key immune genes was performed.

**Figure 7:**
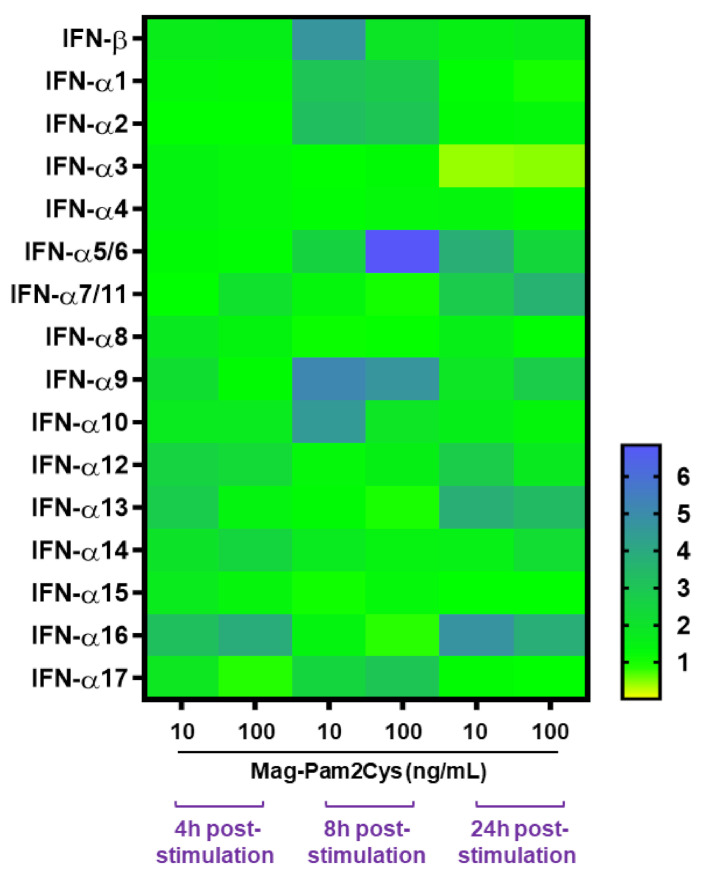
Heatmap of several type I IFNs genes differentially expressed in moMΦ. Porcine moMΦ were left untreated or stimulated with scalar doses of Mag-Pam2Cys (10 or 100 ng/mL). At 4, 8, and 24 h post-stimulation, gene expression levels of IFN-β and several IFN-α subtypes (-α1, -α2, -α3, -α4, -α5/6, -α7/11, -α8, -α9, -α10, -α11, -α12, -α13, -α14, -α15, -α16, -α17) genes were determined using qPCR. At each time point, data were normalized on the values of untreated control and expressed as 2^−ΔΔCt^, with ΔCq = Cq (target gene)—Cq (reference gene), and ΔΔCq = ΔCq (Mag-Pam2Cys-stimulated samples)—ΔCq (untreated sample, moMΦ). Mean data from six independent experiments using different blood donor pigs are shown. The colors in the cells represent the relative magnitude of gene expression. The green color represents the average magnitude of gene expression. The yellow color represents the smallest value, and the brightest blue represents the highest value.

Several type I IFNs were first evaluated. An analysis of IFN-β and IFN-α subtypes gene expression showed that Mag-Pam2Cys induced a statistically significant enhanced expression of IFN-α1, IFN-α2, IFN-α5/6, IFN-α9 at 8 h post-stimulation (Figure 7, Figure S4). A small increase in IFN-α7, -α9, -α12, -α13, -α14, and -α16 gene expression was also observed at 24 h post-stimulation with both Mag-Pam2Cys doses, albeit with only the latter statistically significant (Figure 7, Figure S4).

Th expression of IFN-β was not statistically significantly altered by Mag-Pam2Cys at any time post-stimulation (Figure 7, Figure S5). Interestingly, a marked decrease in IFN-α3 gene expression was observed 24 h post-stimulation with either 10 or 100 ng/mL of Mag-Pam2Cys (Figure 7, Figure S5).

**Figure 8:**
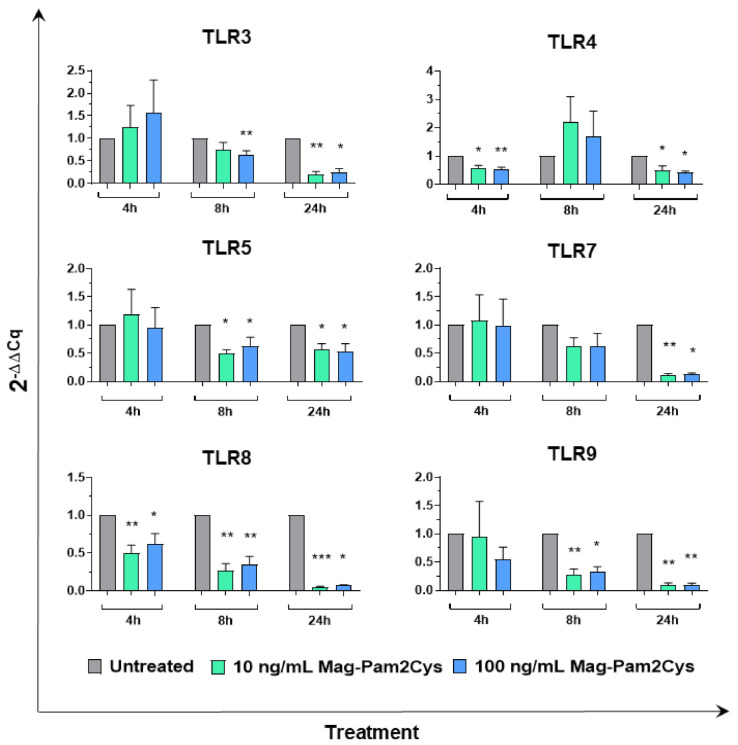
Toll-like receptors’ (TLRs) gene expression in moMΦ following stimulation with Mag-Pam2Cys. MoMΦ were left untreated or stimulated with scalar doses of Mag-Pam2Cys (10 or 100 ng/mL). At 4, 8, and 24 h post-stimulation, gene expression levels of six TLRs (TLR3, TLR4, TLR5, TLR7, TLR8, TLR9) were determined using qPCR. At each time point, data were normalized on the values of untreated control and expressed as 2^−ΔΔCq^, with ΔCq = Cq (target gene)—Cq (reference gene), and ΔΔCq = ΔCq (Mag-Pam2Cys-stimulated samples)—ΔCq (untreated sample, moMΦ). Mean data and SD from six independent experiments using different blood donor pigs are shown. For each time point, values of Mag-Pam2Cys stimulated samples were compared to the corresponding untreated control (moMΦ) using a Kruskal–Wallis multiple comparison test. *** *p* < 0.001, ** *p* < 0.01, * *p* < 0.05.

Modulation on the expression of six TLRs was then evaluated. TLR4 and TLR5 are cell-surface TLRs which recognize bacterial lipopolysaccharide (LPS) or flagellin, respectively, whereas TLR3, TLR7, TLR8, TLR9 are located intracellularly and recognize nucleic acids derived from bacteria and viruses [1,2]. Treatment with either low or high doses of Mag-Pam2Cys induced a statistically significant downregulation of TLR3 (at 8 and 24 h), TLR4 (at 4 and 24 h), TLR5 (at 8 and 24 h), TLR7 (at 24 h), TLR8 (at 4, 8, and 24 h), TLR9 (at 8 and 24 h) (Figure 8).

**Figure 9:**
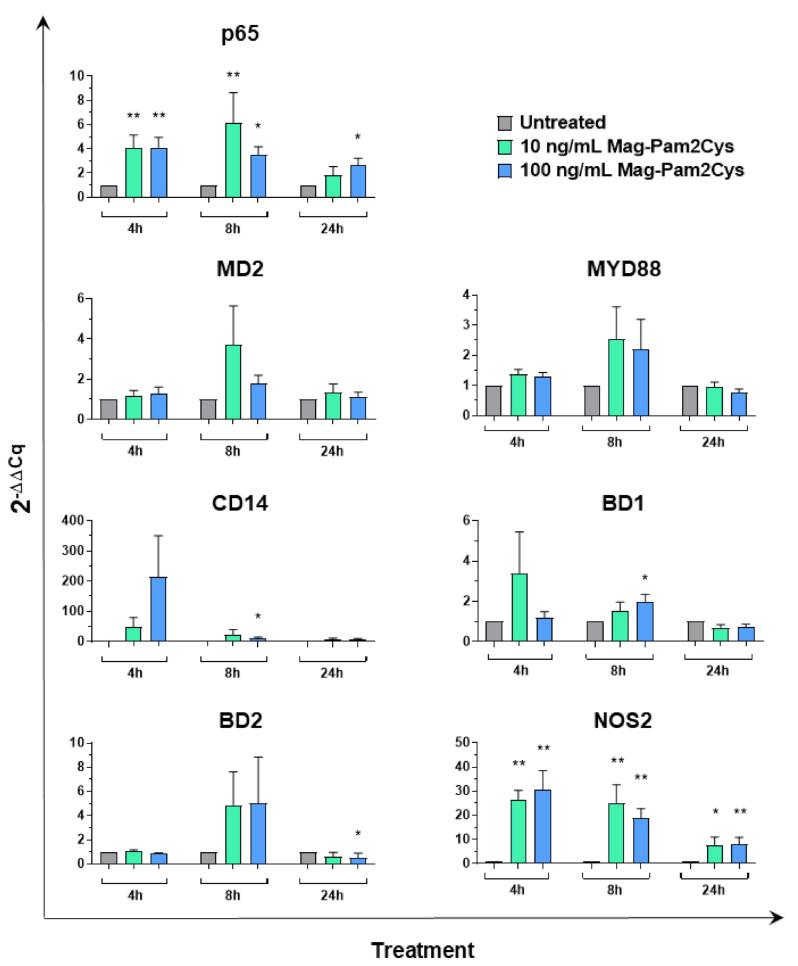
Gene expression of seven key genes of the innate immunity following stimulation with Mag-Pam2Cys. MoMΦ were left untreated or stimulated with scalar doses of Mag-Pam2Cys (10 or 100 ng/mL). At 4, 8, and 24 h post-stimulation, gene expression levels of p65, MYD88, MD2, CD14, BD1, BD2, NOS2 were determined using qPCR. At each time point, data were normalized on the values of untreated control and expressed as 2^−ΔΔCq^, with ΔCq = Cq (target gene)—Cq (reference gene), and ΔΔCq = ΔCq (Mag-Pam2Cys-stimulated samples)—ΔCq (untreated sample, moMΦ). Mean data and SD from six independent experiments using different blood donor pigs are shown. For each time point, values of Mag-Pam2Cys-stimulated samples were compared to the corresponding untreated control (moMΦ) using a Kruskal–Wallis multiple comparison test. ** *p* < 0.01, * *p* < 0.05.

We next investigated the ability of Mag-Pam2Cys to modulate other key genes of innate immunity, such as the adaptor protein myeloid differentiation factor 88 (MYD88) and p65 (a subunit of transcription factor NF-κB), both involved in TLR signaling [43], and four molecules with antimicrobial properties: CD14 and myeloid differentiation factor 2 (MD2), both essential for the recognition of LPS by TLR4 [44], and the host antimicrobial peptides beta defensin 1 (BD1) and 2 (BD2), which exhibit antimicrobial activity against a broad range of bacteria [45,46,47]. The high dose (100 ng/mL) of Mag-Pam2Cys increased expression of p65 (at 4, 8 and 24 h), CD14 (at 8 h), and BD1 (at 8 h) with statistical significance. Enhanced expression of p65 was also observed in moMΦ treated with the low dose (10 ng/mL) of Mag-Pam2Cys (at 4 and 8 h). MYD88 and MD2 expression were not altered by Mag-Pam2Cys at any tested concentration or timepoint (Figure 9). Finally, the effect of Mag-Pam2cys on the expression of nitric oxide synthase 2 (NOS2) was monitored, and we observed that both high and low doses of this TLR2 agonist enhanced NOS2 expression at all time points tested (Figure 9).

### 3.5. Mag-Pam2Cys Ability to Affect moMΦ Response to Recombinant Porcine IL-10

**Figure 10:**
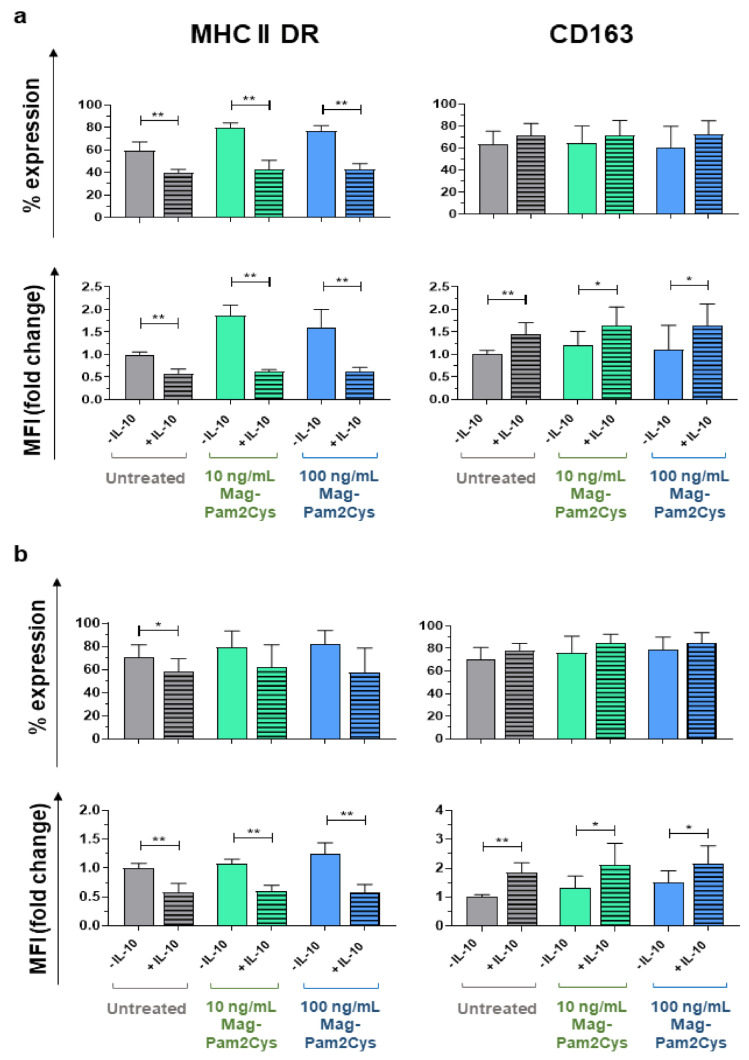
Mag-Pam2Cys modulation of moMΦ response to IL-10 stimulation. Porcine moMΦ were left untreated or stimulated with scalar doses of Mag-Pam2Cys (10 or 100 ng/mL). Simultaneously (**a**) or 24 h later (**b**) recombinant porcine IL-10 (20 ng/mL) was added to well. Surface expression of MHC II DR and CD163 were evaluated by flow cytometry. Percentages of positive cells and mean fluorescence intensity (MFI) data (presented as fold-change relative to the untreated condition, moMΦ) are displayed in both panels. For both Panels (**a**,**b**), mean data and SD from three independent experiments using different blood donor pigs are presented. Values of stimulated samples were compared to the corresponding untreated control (moMΦ) using a one-way ANOVA followed by a Dunnett’s multiple comparison test. ** *p* < 0.01, * *p* < 0.05.

To explore the effect of Mag-Pam2Cys on porcine moMΦ plasticity further, we evaluated its ability to modulate macrophage polarization induced by IL-10. We recently described that IL-10 polarizes porcine moMΦ towards an immunosuppressive phenotype (moM(IL-10)), characterized by a strong impairment of their ability to respond to either TLR2 or TLR4 agonists [19]. Porcine moM(IL-10) were also depicted by MHC II DR downregulation and enhanced surface expression of CD163 [19]. We evaluated whether Mag-Pam2Cys-induced activation impacted MHC II DR downregulation and CD163 upregulation triggered by recombinant porcine IL-10 (Figure 10). Treatment of moMΦ with Mag-Pam2Cys either 24 h before (Figure 10a) or simultaneously (Figure 10b) with IL-10 treatment did not alter the downregulation of MHC II DR or upregulation CD163.

## 4. Discussion

Macrophages play a central role in the innate immune response to both infectious and non-infectious stressors. They respond to different agonists modifying their phenotype and functions [48]. Macrophages polarized towards a M1 phenotype enhance defenses to intracellular pathogens and promote tumor regression, whereas alternatively activated macrophages (M2) are mainly associated with wound-healing and tissue repair [48]. Switching macrophages from a pro-tumor type (M2 phenotype) to an anti-tumor state (M1 phenotype) is a promising strategy in cancer immunotherapy [7]. Macrophages are equipped with a vast array of PRRs, including TLRs [21]. Among TLRs, TLR2 initiates potent immune responses by recognizing diacylated and triacylated lipopeptides, forming heterodimers with either TLR6 or TLR1, respectively [49]. We previously observed that Mag-Pam2Cys, a synthetic diacylated lipopeptide derived from a surface lipoprotein of *M. agalactiae*, induced a strong cytokine response from porcine moMΦ [19,20]; thus, in this study, we further investigated the genomic and functional impact of this lipopeptide on porcine moMΦ.

We observed that Mag-Pam2Cys enhanced the surface expression of molecules involved in antigen presentation (MHC class I and II) and the IL-2 receptor CD25, increased moMΦ phagocytotic activities, and enhanced the expression and release of IL-12 and proinflammatory cytokines (IL-1α, IL-1β, IL-6, TNF-α) in a dose-dependent manner. Our results are in accordance with that previously observed in humans with MALP-2: the stimulation of human monocytes in vitro resulted in a sustained increased expression and release of cytokines (IL-6, TNF-α) and chemokines (IL-8, GRO-α, MCP-1, MIP-1α, and MIP-1β) [50]. In the same study, a weak induction of the anti-inflammatory IL-10 was also observed [50], and this is in accordance with our results with porcine moMΦ. IL-10 induction or release are not hallmarks of M2 polarization in swine [19,34] and it was previously reported by us and others that stimulation of porcine moMΦ with IFN-γ and LPS (classical M1 activation stimuli) induced a low, but statistically significant, release of IL-10 [34,46]. Overall, our data suggest that Mag-Pam2Cys polarized porcine macrophages toward a M1-like phenotype, which in pigs is associated with increased expression of MHC I, MHC II DR, CD25, co-stimulatory molecules, and the release of proinflammatory cytokines [34,42,51].

To evaluate the polarizing effect of Mag-Pam2Cys on pig macrophages better, we directly compared the impact of a high dose (100 ng/mL) with the classical M1 polarizing factors (100 ng/mL IFN-γ and 100 ng/mL LPS). MoMΦ stimulated with Mag-Pam2Cys presented a lower expression of CD25, CD169, MHC II DR and a higher expression of CD14 compared to *bona fide* moM1. MoM1 were characterized by a higher release of IL-12, whereas moMΦ stimulated with Mag-Pam2Cys triggered a higher release of several proinflammatory cytokines (IL-1α, IL-1β, IL-6). These results highlight the plasticity of macrophages, which change their phenotype and function in response to different stimuli [48]. Indeed, it should be more appropriate to use a nomenclature linked to the activator(s) used instead of M1/M2, as suggested by Murray et al. [52]. To obtain a better understanding of the breadth of effects of Mag-Pam2Cys on porcine moMΦ, we analyzed its modulation of a panel of key innate immunity genes. Type I IFNs are a large family of antiviral proteins [53]. The porcine type I IFN family is complex and composed of at least 39 functional genes: one IFN-β, -αω, -ε, and -κ; 17 IFN-α; 11 IFN-δ; and 7 IFN-ω subtypes [54]. Despite their high structural homology, porcine IFN-α subtypes exhibit different anti-inflammatory, immunomodulatory, and antiviral activities against viruses, such as pseudorabies virus, PRRSV, classical swine fever virus (CSFV), and foot-and-mouth disease virus (FMDV) [53,54,55,56]. Thus, differences in the production of IFN-α subtypes might affect the endurance and intensity of an antiviral response. Mag-Pam2Cys induced a modest, but statistically significant, enhanced expression of IFN-α1, IFN-α2, IFN-α5/6, IFN-α9, and IFN-α16. These porcine IFNs exhibited direct antiviral activities towards many viruses, including PRRSV [54], CSFV [57], and pseudorabies [53], and presented immunomodulatory properties (inducing MHC I and MHC II upregulation) [53]. The induction of several type I IFNs, alongside proinflammatory cytokines, suggests that Mag-Pam2Cys would promote host defense responses able to protect the host from microbial infection. However, the expression of IFN-β and other IFN-α subtypes was not statistically significantly altered by Mag-Pam2Cys at any time points, and, most interestingly, a marked decrease in IFN-α3 gene expression was observed 24 h post-stimulation. Our results showed that type I IFNs were differently modulated upon stimulation with Mag-Pam2Cys. Further studies are needed to improve our understanding of the biological activities of individual type I IFNs and to appreciate fully how differences in their expression could influence the quality of antiviral responses.

We next specifically addressed how Mag-Pam2Cys modulated the expression of six TLRs and molecules involved in TLRs signaling. We observed that Mag-Pam2Cys treatment downregulated the expression of all the TLRs tested (TLR3, TLR4, TLR5, TLR7, TLR8, TLR9). Downregulation of TLR-4 and TLR8 was observed as soon as 4 h post-stimulation, whereas all the tested TLRs genes were drastically downregulated 24 h post-stimulation. A potential concern with stimulating macrophages through activation of TLRs is the induction of inappropriate inflammatory responses [3]; thus, the downregulated expression of other TLRs by Mag-Pam2Cys might present advantages in vivo. PAMP engagement by TLRs triggers several intracellular signaling cascades involving adapter molecule MyD88 and NF-κB, culminating in cytokine production and release [3,6]. The downregulated expression of these TLRs may represent protective mechanisms, considering that a tight regulation of this process is essential to avoid the development of pathological inflammatory responses or even autoimmunity [58].

The adaptor proteins MYD88 and the transcription factor NF-κB are both involved in the TLR2 intracellular signaling. Phosphorylation of p65 enhances the NF-κB transcriptional response and thus induction of inflammatory cytokine genes [42]. We observed that this TLR2 agonist induced a strong increase in p65 gene expression, even at low doses, and this is in accordance with the observed enhanced expression and release of proinflammatory cytokines. Nevertheless, MYD88 expression was not modulated by Mag-Pam2Cys. We speculate that this null modulation of this adaptor protein might be the result of both positive and negative signals. As stated above, TLR signaling must be tightly regulated to prevent or terminate an excessive immune response. We then evaluated Mag-Pam2Cys’ impact on the gene expression of four molecules with antimicrobial properties: CD14 and MD2, BD1, and BD2 [43,44,45,46]. We observed that high doses of the diacylated lipopeptide under investigation increased the expression of either CD14 or BD1 with statistical significance, indicating that Mag-Pam2Cys stimulation triggered the enhancement of macrophage antimicrobial activities. Finally, modulation of NOS2 expression by this TLR2 agonist was evaluated. NOS2 encodes for the enzyme inducible nitric oxide synthase (iNOS), which generates nitic oxide (NO) from arginine [59]. NOS2 expression increased over time following Mag-Pam2Cys stimulation, further denoting the enhanced macrophage microbicidal functions promoted by this TLR2 agonist. Interestingly, iNOS expression is a hallmark of M1 polarization in many species [59], but not in swine. A previous study described that M1 polarization in pigs did not result in NO production or NOS2 upregulation [42], suggesting that future studies are needed to define macrophage polarization in pigs better.

Considering these observations, we speculate that administration of Mag-Pam2Cys in vivo might lead to macrophage activation with the release of proinflammatory cytokines, resulting in the recruitment of immune cells in situ and improving host defense against invading pathogens, as described in mice after intratracheal MALP-2 application or intranasal PEG-Pam2Cys administration [9,14]. In fact, intra-tracheal administration of MALP-2 to wild type (WT), but not TLR2-deficent, mice evoked enhanced proinflammatory cytokine and chemokine release, along with leukocyte recruitment into the lung [9]. Moreover, application of MALP-2 24 h before intranasal infection with *S. pneumonia* resulted in increased survival, reduced bacteremia, and improved bacterial clearance in lung parenchyma in WT mice [9]. As mentioned above, intranasal administration of the synthetic PEG-Pam2Cys to mice led to reduced viral transmission rates following influenza infection, and promoted the development of adaptive immune responses, measured by the induction of influenza A virus-specific CD8+ T cells [14,15].

Overall, our results showed that Mag-Pam2Cys polarizes porcine macrophages toward a proinflammatory, antimicrobial phenotype [42,60]. Thus, we evaluated its ability to affect polarization triggered by the immunosuppressive IL-10 [21]. We recently described that IL-10 triggered MHC II DR downregulation and enhanced surface expression of CD163 on pig moMΦ [19]. Interestingly, Mag-Pam2Cys did not alter IL-10-induced MHC II DR downregulation or CD163 upregulation at any of the concentrations tested, either when administered 24 h before or in concomitance with porcine recombinant IL-10. The null interference of Mag-Pam2Cys with the activity of the immunosuppressive IL-10 stimulation is promising for the potential use of Mag-Pam2Cys as an immunotherapeutic agent, considering that IL-10 might regulate the development of exacerbated immune responses in vivo. Macrophages are characterized by remarkable plasticity [48]; thus, future studies should address how this TLR2 agonist affects macrophage polarization triggered by other immunosuppressive factors (as TGF-β or glucocorticoids), or M2a factors (IL-4, IL-13), to evaluate its use as an immunotherapeutic agent better.

## 5. Conclusions

Herein, we have provided a detailed characterization of the effects of a synthetic diacylated mycoplasma-derived pam2cys lipopeptide, Mag-Pam2Cys, on porcine macrophages. We found that this TLR2 agonist induced enhanced expression of activation markers, increased phagocytotic activity, and the release of IL-12 and proinflammatory cytokines. Mag-Pam2Cys also induced expression of p65, CD14, BD1, NOS2, and several type I IFNs, indicating a polarization of macrophages towards a proinflammatory, antimicrobial phenotype. Nevertheless Mag-Pam2Cys negatively modulated TLR3, 4, 5, 7, 8, 9 gene expression and did not interfere with macrophage polarization induced by the immunosuppressive IL-10, suggesting that the inflammatory response induced by Mag-Pam2Cys can be regulated. This study lays the foundation for the further evaluation of this TLR-2 agonist in vivo as an immunomodulatory agent for pigs and other species.

## Data Availability

The data presented in the study are available on request from the corresponding author.

## Supplementary Materials

The following are available online at https://www.mdpi.com/article/10.3390/vaccines9070692/s1, Figure S1. Study design. Figure S2. No morphological alterations of porcine moMΦ following Mag-Pam2Cys stimulation. Figure S3. Representative dot plots of untreated or Mag-Pam2Cys-stimulated porcine moMΦ. Figure S4. Cytokine gene expression in moMΦ following stimulation with Mag-Pam2Cys. Figure S5. Type I IFN gene expression in moMΦ following stimulation with Mag-Pam2Cys. Table S1. Antibodies used for flow cytometry. Table S2. Oligonucleotide Primer Sets for Evagreen qRT Real-Time PCR.
