# Supplementary material for: Targeting Toll-Like Receptor 2: Polarization of Porcine Macrophages by a Mycoplasma-Derived Pam2cys Lipopeptide
Source: Vaccines (Basel). 2021 Jun 23;9(7):692. doi: 10.3390/vaccines9070692 (PMC8310132; doi:10.3390/vaccines9070692)
Supplement: Supplementary file 1 [file vaccines-09-00692-s001.zip › vaccines-1243970-supplementary.pdf]

Table S1. Antibodies used for flow cytometry.

Antibody	Reactivity	Clone	Isotype	Conjugate	Concentration (mg/mL)	Working dilution
**Primary Antibodies**						
CD14	Human	Tuk4	IgG2a	Per-CP	ND	1/4
MHC II DR	Pig	2E9/13	IgG2b	FITC	1	1/25
MHC I	Pig	JM1E3	IgG1	-	1	1/40
CD25	Pig	K231.3B2	IgG1	-	1	1/25
CD163	Pig	2A10/11	IgG1	PE	ND	1/4
CD169	Pig	MCA2316F	IgG1	FITC	0.1	1/4
**Secondary Antibodies**						
Anti-IgG1	Mouse	A85-1	IgG1	BV421	0.2	1/25

ND: not determined.

Table S2. Oligonucleotide Primer Sets for Evagreen qRT Real-Time PCR.

Gene	Sequences	Reference / Accession
IL 1β	F: 5′-AATTCGAGTCTGCCCTGTACCC-3′<br>R: 5′-TGGTGAAGTCGGTTATATCTTGGC-3′	[56]
IL6	F: 5′-CAGAGATTTTGCCGAGGATG-3′<br>R: 5′-TGGCTACTGCCTTCCCTACC-3′	[56]
IL10	F: 5′-AGCCAGCATTAAGTCTGAGAA-3<br>R: 5′-CCTCTCTTGGAGCTTGCTAA-3′	[19]
IL 12p40	F: 5'-TCAGGGACATCATCAAACCA-3'<br>R: 5'-GAACACCAAACATCAGGGAAA-3'	[19]
TNFα	F: 5′-TGCCTACTGCACTTCGAGGTTATC-3′<br>R: 5′-GTGGGCGACGGGCTTATCTG -3′	[57]
βD1	F: 5′CTGTTAGCTGCTTAAGGAATAAAGGC-3′<br>R: 5′-TGCCACAGGTGCCGATCT-3′	[56]
βD2	F: 5′-CCAGAGGTCCGACCACTA-3′<br>R: 5′-GGTCCCTTCAATCCTGTT-3′	[56]
MyD88	F: 5′-GCAGCTGGAACAGACCAACT-3′<br>R: 5′-GTGCCAGGCAGGACATCT-3′	[56]
CD14	F: 5′-TGCCAAATAGACGACGAAGA-3′<br>R: 5′-ACGACACATTACGGAGTCTGA-3′	[56]
MD2	F: 5′-TGCAATTCCTCTGATGCAAG-3′<br>R: 5′-CCACCATATTCTCGGCAAAT-3′	[56]
NFkB/p65	F: 5′-CGAGAGGAGCACGGATACCA-3′<br>R: 5′-GCCCCGTGTAGCCATTGA-3′	[56]
IFNβ	F: 5′-AGTTGCCTGGGACTCCTCAA-3′<br>R: 5′-CCTCAGGGACCTCGAAGTTCAT-3′	[58]
IFNα1	F: 5'-GGCTCTGGTGCATGAGATGC-3'<br>R: 5'-GCCTTCTTCCTGAATCTGTCTCA-3'	[59]
IFNα2	F: 5′-CAACCTCAGCCTTCCTCACA-3′<br>R: 5′-GAATCTGTCTCACAGGTTTC-3′	[59]
IFNα3	F: 5′-TGAGGAGAATCTCCCCT-3′<br>R: 5′-CTTCCTGAATCTGTCTCA-3′	[59]
IFNα4	F: 5′-TCTCTGGGCTGCGACCCGGT-3′<br>R: 5-CCTCCTGAGTCTGTCTTG-3	[59]
IFNα5/6	F: 5′-GCACAAATGAGGAGAATATCT-3′<br>R: 5′-CCTCCTGAGTCTGTCTTG-3′	[59]
IFNα7/11	F: 5′-GGGACTTTGGATCCCCTCAT-3′<br>R: 5′-GTGGAGGAAGAGAAGGATG-3′	[59]

IFNα8	F: 5′-AACCTCAGCCTTCCTCACG-3′<br>R: 5′-GTCTGTCTTGCAGGTTTG-3′	[59]
IFNα9	F: 5′-GTGCTGCTCAGCTGCAAG-3′<br>R: 5′-AGTCCTCCTCCAGCAGGGGC-3′	[59]
IFNα10	F: 5′-AAAGGACTTTGGATTCCCCC-3′<br>R: 5′-ATGACACAGGCTTCCAGGTC-3′	[59]
IFNα12	F: 5′-CCTCAGCCTTCCTCACGGT-3′<br>R: 5′-CTCATGACTTCTGCCCTGAT-3′	[59]
IFNα13	F: 5′-ATCCTCAGCCCTCCTCAC-3′<br>R: 5′-ATCCAAAGTCCCTTCTGT-3′	[59]
IFNα14	F: 5′-TCAACTCTCCTCACGGTC-3′<br>R: 5′-GTCCAGGCAGGAGAAGG-3′	[59]
IFNα15	F: 5′-CTCTCTGGGCTGCGACCT-3′<br>R: 5′-CTCCTTCCTCCTGAGTCTGTC-3′	[59]
IFNα16	F′: 5-GTTCAGACCCACAGCCTG-3′<br>R′: 5-TCCTCCTGAGTCTGTCTTGC-3′	[59]
IFNα17	F: 5′-CTGCCTCAGACCCATAGC-3′<br>R: 5′-CTCTTCTCTTGCAGATAGAGGG-3′	[59]
TLR3	F: 5′-TGAAGAACTTGATTTCCTTGGCA-3′<br>R: 5′- GGCATGAAAACACCCTGGAG-3′	NM_001097444
TLR4	F: 5'-TGGCAGTTTCTGAGGAGTCATG-3'<br>R: 5'-CCGCAGCAGGGACTTCTC-3’	[53]
TLR5	F: 5’-TCAAAGATCCTGACCATCACA-3’<br>R: 5’-CCAGCTGTATCAGGGAGCTT-3’	[53]
TLR7	F: 5’-GTGGAAATTGCCCTCGTTGT-3’<br>R: 5’-GATGGATCTGTAGGGGAGCA-3’	NM_001097434
TLR8	F: 5′-AAGACAACCAGTTACGTGAAATACC-3′<br>R: 5′-GGGTGTTAAAAGATAATGACAGCAC-3′	[60]
TLR9	F: 5′-AGGACTTCATGCCAAACTGC-3′<br>R: 5′-CGAGCAAACATCTCCGACTG-3′	NM_213958
NOS2	F: 5'-CGT TAT GCC ACC AAC AAT GG-3'<br>R: 5'-AGA CCC GGA AGT CGT GCT T-3'	[37]
B2M	F: 5′ CGCCCCAGATTGAAATTGATTTGC-3′<br>R: 5′ GCTATACTGATCCACAGCGTTAGG-3′	[35]
GAPDH	F: 5′-ACCCAGAAGACTGTGGATGG-3′<br>R: 5′-ACGCCTGCTTCACCACCTTC-3′	[35]
HPTR1	F: 5′-TTCCTTGGTCAAGCAGCATAATCC-3′<br>R: 5′-AAGGGCATAGCCTACCACAAAC-3′	[35]

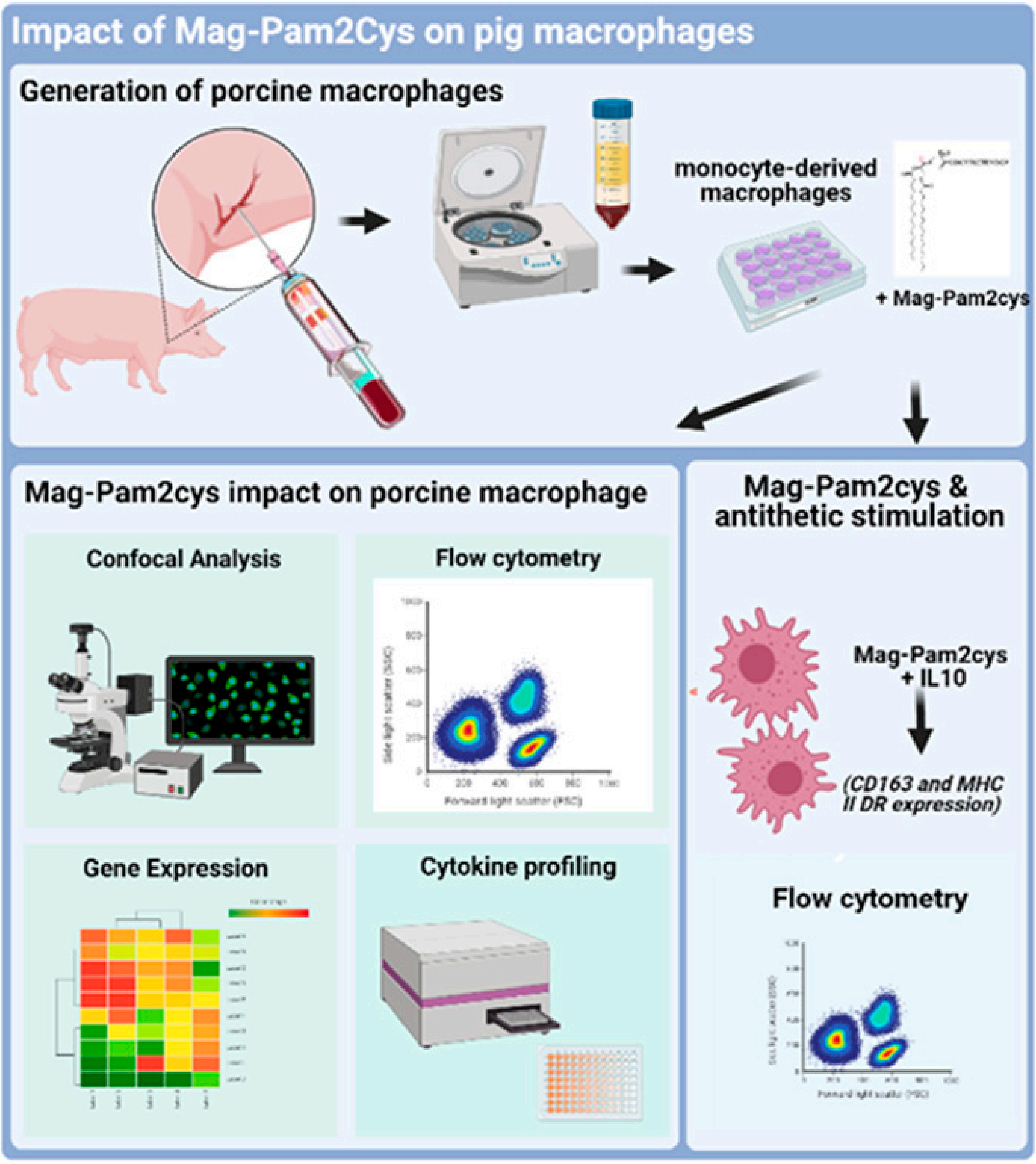

Figure S1. Study design.

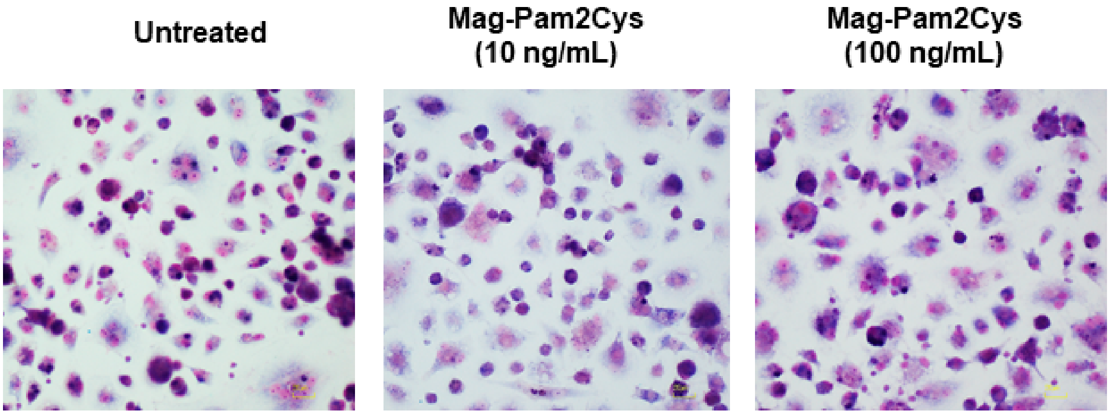

Figure S2. No morphological alterations of porcine moMΦ following Mag-Pam2Cys stimulation. Porcine moMΦ were stimulated scalar doses of Mag-Pam2Cys (10 or 100 ng/mL) or left untreated. 24 h post-stimulation, moMΦ were morphologically evaluated though May-Grunwald-Giemsa staining. Images of representative moMΦ, one from each condition (untreated, 10 or 100 ng/mL of Mag-Pam2Cys) are presented. Scale bar, 20 μm.

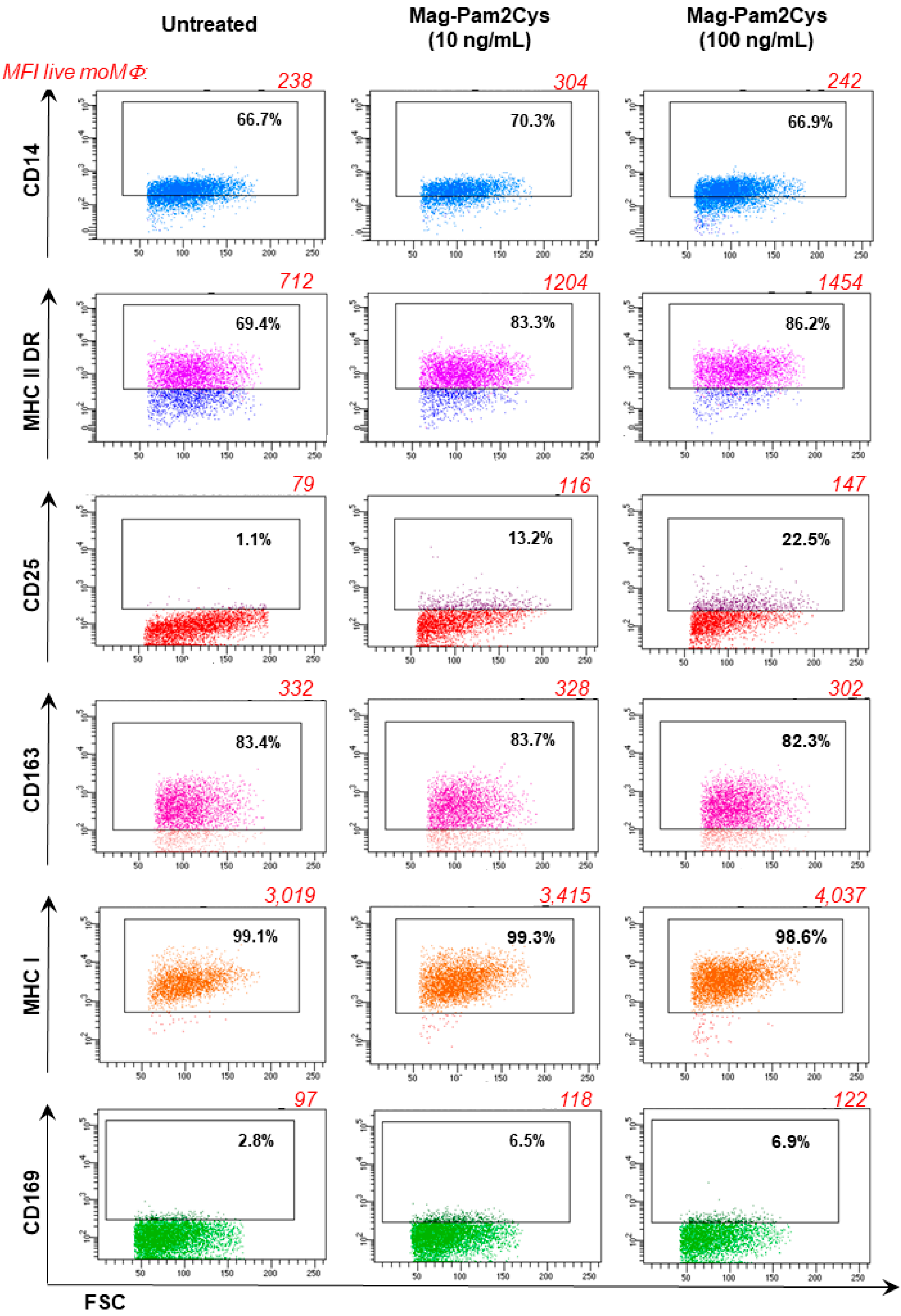

Figure S3. Representative dot plots of untreated and Mag-Pam2Cys stimulated moMΦ. Porcine moMΦ were left untreated or stimulated with scalar doses of Mag-Pam2Cys (10 or 100 ng/mL). 24 h post-stimulation with scalar doses of Mag-Pam2Cys (10 or 100 ng/mL), surface expression of CD14, MHC II DR, CD25, CD163, MHC I, and CD169 were assessed by flow cytometry. Representative dot plots for each treatment conditions (untreated, 10 ng/mL Mag-Pam2Cys, 100 ng/mL Mag-Pam2Cys) and markers are displayed, with the percentages of positive cells shown and the mean fluorescence intensity (MFI) of live moMΦ presented above each plots.

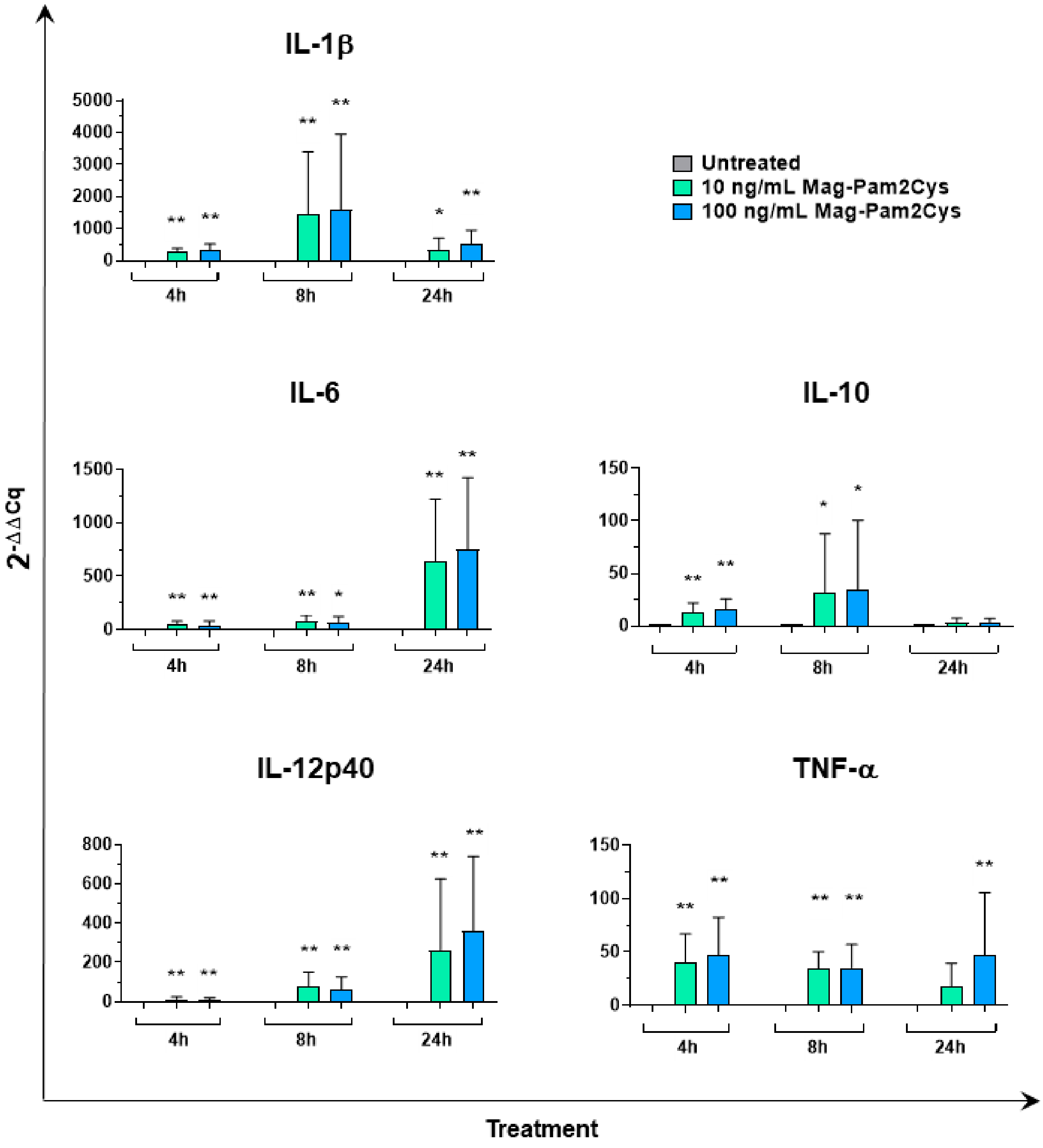

Figure S4. Cytokine gene expression in moMΦ following stimulation with Mag-Pam2Cys. moMΦ were left untreated or stimulated with scalar doses of Mag-Pam2Cys (10 or 100 ng/mL). At 4, 8, and 24 h post-stimulation, gene expression levels of IL-1β, IL-6, IL-10, IL-12, TNF-α were determined using qPCR. At each time point, data were normalized on the values of un-treated control and expressed as 2−ΔΔCq, with ΔCq = Cq (target gene) − Cq (reference gene), and ΔΔCq = ΔCq (Mag-Pam2Cys-stimulated samples) − ΔCq (un-treated sample, moMΦ). Mean data and SD from six independent experiments using different blood donor pigs are shown. For each time point, values of Mag-Pam2Cys stimulated samples were compared to the corresponding untreated control (moMΦ) using a Kruskal–Wallis multiple comparison test. ** $p < 0.01$, * $p < 0.05$.

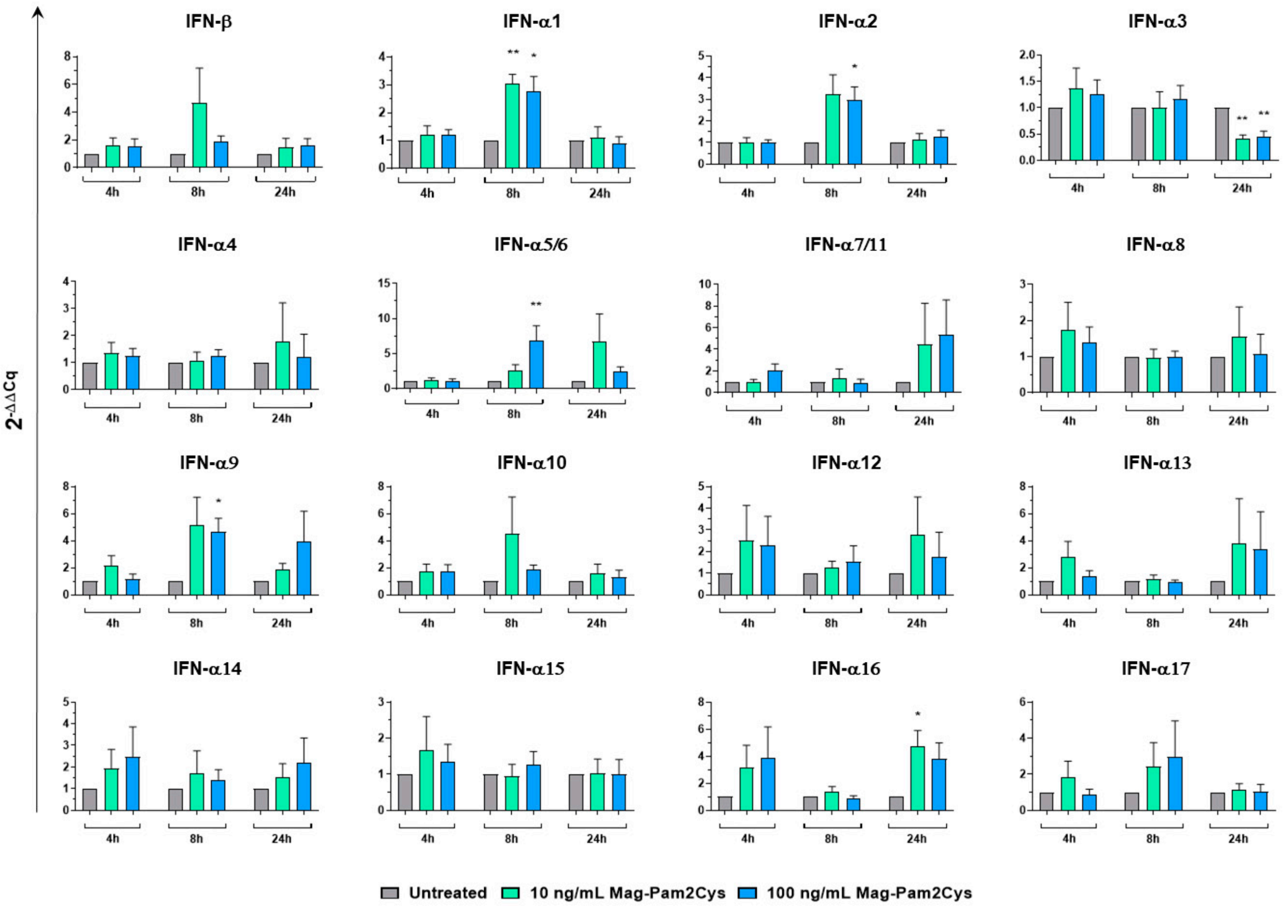

Figure S5. Type I IFN gene expression in moMΦ following stimulation with Mag-Pam2Cys. moMΦ were left untreated or stimulated with scalar doses of Mag-Pam2Cys (10 or 100 ng/mL). At 4, 8, and 24 h post-stimulation, gene expression levels of IFN-β and six IFN-α subtypes (-α1, -α2, -α3, -α4, -α5/6, -α7/11, -α8, -α9, -α10, -α11, -α12, -α13, -α14, -α15, -α16, -α17) were determined using qPCR. At each time point, data were normalized on the values of un-treated control and expressed as $2^{-\Delta\Delta Cq}$, with ΔCq = Cq (target gene) − Cq (reference gene), and ΔΔCq = ΔCq (Mag-Pam2Cys-stimulated samples) − ΔCq (un-treated sample, moMΦ). Mean data and SD from six independent experiments using different blood donor pigs are shown. For each time point, values of Mag-Pam2Cys stimulated samples were compared to the corresponding untreated control (moMΦ) using a Kruskal–Wallis multiple comparison test. ** $p < 0.01$, * $p < 0.05$.
